# Total Arterial Revascularization - A Fascinating Approach Still Not Widely Accepted

**DOI:** 10.21470/1678-9741-2021-0238

**Published:** 2021

**Authors:** Stefano D’Alessandro, Francesco Maestri, Francesco Nicolini, Francesco Formica

**Affiliations:** 1 Cardiac Surgery Unit, Vito Fazzi Hospital, Lecce, Italy; 2 Cardiac Surgery Unit, Parma General Hospital, Department of Medicine and Surgery, University of Parma, Italy.

Total arterial myocardial revascularization (TAR) in the context of coronary patients with three-vessel disease is a surgical approach as fascinating as rarely used. The use of TAR is not over 15% worldwide, although TAR is one of the more investigated topics in cardiac surgery and there is a consistent evidence suggesting that patients who receive TAR have better postoperative outcome and superior life expectancy^[1]^. However, TAR is still not widely accepted.

In their meta-analysis, Rayol et al.^[2]^ aimed to analyze the impact of TAR on long-term survival. They selected 14 articles and obtained a pool of 22,476 patients, 8,941 received TAR and 13,805 underwent no-TAR surgery. Both sensitivity and meta-regression analysis did not affect the results of meta-analysis. The hazard ratio (0.676) (95% confidence interval 0.586-0.779; *P*<0.0001) for long-term survival was significantly in favour of TAR procedure.

Some of the studies included in the meta-analysis reported data and results regarding the use of radial artery (RA) in addition to bilateral internal thoracic arteries (BITA), and this combination represents a valid approach to obtain TAR in three-vessel patients. We recently reported optimal long-term results (up to 15 years) in patients who underwent either BITA + RA or BITA plus saphenous vein (SV), although we failed to demonstrate a significant better long-term benefit of RA over the SV graft^[3]^. A recent meta-analysis by Di Mauro reported no differences between the two surgical treatments^[4]^. Indeed, the choice of arterial grafts as third arterial conduit is a matter of debate. The 2018 European Society of Cardiology (ESC) and the 2016 Society of Thoracic Surgeon (or STS) guidelines cited BITA as a Class IIA indication in patients with low risk of sternal wound infection, whereas RA receives a Class I indication over SV in patients with high grade of coronary artery stenosis in the 2018 ESC guidelines^[5,6]^. Rayol et al.^[2]^ have focused on some of the barriers which discourage TAR, such as the prone to the spasm and the higher technical difficulties. These authors’ comments are completely shareable. Furthermore, it must be considered that more time is needed to perform TAR. Data from the Arterial Revascularization Trial (or ART) of unplanned conversion from BITA to a single internal thoracic artery graft demonstrated the technical challenge precluding a BITA grafting in a group of surgeons selected for their expertise in that approach^[7]^. Furthermore, during the last 10 years, the attention of surgeons has shifted towards more sophisticated and attractive surgical treatments, such as minimally invasive valve surgery, robotic surgery, and complex aortic surgery. Least but not last, the latest guidelines have given many spaces to the percutaneous treatment of coronary disease, thus reducing the number of coronary patients to be referred for surgery. All these factors have probably generated in many surgeons a lack of interest towards complex coronary surgery, thus preferring the more traditional and faster approach with a single arterial graft rather than TAR. This could explain why, despite the encouraging results, TAR has still few considerations and is applied routinely by a few centers and why the role of meta-analysis, such as this one, in disseminating the favorable long-term results of TAR is relevant.

One of the limitations of Rayol’s study is the lack of data and results regarding the coronary target of RA grafting, the grade of stenosis, and the usage of TAR in older and sicker patients. Nevertheless, we are aware of the scarcity of such data and results in the literature, especially regarding the last two scenario and the still unclear long-term outcome (more than 15 years) of TAR, due to few randomized trails and observational multicenter studies. Lastly, TAR should be strongly encouraged even revising the guidelines to emphasize the role of referral centers offering a high likelihood of TAR and low mortality in three-vessel coronary patients.
